# Illusory Motion Reversal in Touch

**DOI:** 10.3389/fnins.2019.00605

**Published:** 2019-06-14

**Authors:** Yu-Chun Hsu, Chun-I Yeh, Jian-Jia Huang, Chang-Hung Hung, Chou Po Hung, Yu-Cheng Pei

**Affiliations:** ^1^Institute of Neuroscience, National Yang-Ming University, Taipei, Taiwan; ^2^Neurobiology and Cognitive Science Center, National Taiwan University, Taipei, Taiwan; ^3^Graduate Institute of Brain and Mind Sciences, College of Medicine, National Taiwan University, Taipei, Taiwan; ^4^Department of Psychology, College of Science, National Taiwan University, Taipei, Taiwan; ^5^Department of Physical Medicine and Rehabilitation, Linkou Chang Gung Memorial Hospital, Taoyuan, Taiwan; ^6^Department of Medicine, College of Medicine, Chang Gung University, Taoyuan, Taiwan; ^7^Center of Vascularized Tissue Allograft, Linkou Chang Gung Memorial Hospital, Taoyuan, Taiwan; ^8^U.S. Army, CCDC Army Research Laboratory, Aberdeen, MD, United States; ^9^Department of Neuroscience, Georgetown University Medical Center, Washington, DC, United States; ^10^Healthy Aging Research Center, Chang Gung University, Taoyuan, Taiwan

**Keywords:** touch, illusion, somatosensory, perceptual rivalry, perception

## Abstract

Psychophysical visual experiments have shown illusory motion reversal (IMR), in which the perceived direction of motion is the opposite of its actual direction. The tactile form of this illusion has also been reported. However, it remains unclear which stimulus characteristics affect the magnitude of IMR. We closely examined the effect of stimulus characteristics on IMR by presenting moving sinusoid gratings and random-dot patterns to 10 participants’ fingerpads at different spatial periods, speeds, and indentation depths. All participants perceived a motion direction opposite to the veridical direction some of the time. The illusion was more prevalent at spatial periods of 1 and 2 mm and at extreme speeds of 20 and 320 mm/s. We observed stronger IMR for gratings and much weaker IMR for a random-dot pattern, indicating that edge orientation might be a major contributor to this illusion. These results show that the optimal parameters for IMR are consistent with the characteristics of motion-selective neurons in the somatosensory cortex, as most of these neurons are also orientation-selective. We speculate that these neurons could be the neural substrate that accounts for tactile IMR.

## Introduction

Illusory motion reversal (IMR) is a phenomenon in which the perceived motion direction is the opposite of its physical direction. The most renowned example of visual IMR is the wagon-wheel illusion, which occurs when observing a circular array of bars printed on a rotating disc (Schouten, 1967; Purves et al., 1996). The existence of visual IMR reveals several properties of the neuronal mechanisms that process visual motion information, and this study aims to examine IMR for the tactile system.

Tactile IMR was first reported by Holcombe and Seizova-Cajic (2008) as perceived motion-direction reversal when participants grasped a rotating cylinder. However, they only characterized the accuracy of motion-direction discrimination, without investigating its underlying mechanism. To our knowledge, no study has yet linked tactile IMR to neurophysiological properties of mechanoreceptors or sensory neurons.

The somatosensory system encodes the spatio-temporal information of touch through a hierarchical organization. Mechanical energy is transduced by receptors in the skin into electrical impulses that ascend to neurons in the primary somatosensory cortex (S1), yielding orientation and motion-direction information that is fundamental for shape and motion perception. In glabrous skin, touch is mediated by four types of mechanoreceptors (Darian-Smith and Oke, 1980; Goodwin and Morley, 1987), each sensitive to a specific type of somatosensory input or a specific frequency band. Both slowly adapting type 1 (SA1) and rapidly adapting (RA) afferent receptors encode information about stimulus orientation and support orientation discrimination ability (Pruszynski and Johansson, 2014). Spatial information is refined in S1, where a majority of neurons show orientation and motion-direction selectivity (Olausson et al., 2000; Bensmaia et al., 2008; Pei et al., 2010). Although motion-selective spiking responses have been characterized in S1, the neuronal mechanisms that yield orientation and motion-direction tuning remain unclear (Pei et al., 2008, 2011; Pei and Bensmaia, 2014). Among motion-direction selective neurons in S1, a majority is also orientation selective, and their preferred motion direction is orthogonal to their preferred orientation (Pei et al., 2010). This well-known phenomenon of orthogonality, known as the “aperture effect,” is thought to be due to the inherent ambiguity of motion direction and speed information available to S1 neurons, because of their small receptive fields. For example, when sensed through a round aperture, the perceived motion direction is always orthogonal to the orientation of the edge, independent of the veridical motion direction. Understanding the psychophysical characteristics of IMR could thus shed light on the neuronal mechanisms that account for tactile feature processing.

We tested several hypotheses about the phenomenon of tactile IMR, namely that its perception would depend on the spatio-temporal parameters, indentation depth, and shape of the stimuli. Each of the SA1, RA1, and Pacinian mechanoreceptors has a specific preferred temporal frequency (Johnson, 2001). For the dependence on spatio-temporal parameters, we hypothesized that if inputs from specific types of mechanoreceptors drive tactile IMR, the magnitude of IMR will match their properties by showing a frequency preference. If IMR is mediated by cortical processing, e.g., in S1, it will have preferences in both speed and direction of motion with respect to the skin, and it will exhibit directional anisotropy (Essock et al., 1992, 1997; Olausson and Norrsell, 1993; Keyson and Moutsma, 1995; Drewing et al., 2005), in which the effect is stronger in specific motion directions. Second, we hypothesized that an increase in indentation depth will enhance the performance of directional discrimination and decrease IMR. This expectation of dependency is supported by a previous report that the indentation depth of a stimulus is correlated to the signal strength of SA1 and RA inputs (Johnson and Hsiao, 1992). Third, our test also addressed an issue in the visual IMR literature on the shape dependency of IMR, specifically, whether IMR can only be induced by periodic stimulus patterns as claimed by several reports (Purves et al., 1996; Simpson et al., 2005; VanRullen et al., 2005), or whether non-periodic random-dot patterns could also induce IMR (Kline and Eagleman, 2008). We hypothesized that orientation information (i.e., the grating) is necessary for the induction of tactile IMR, and that tactile IMR will be abolished by random-dot patterns. To test our hypotheses, we presented moving sinusoid gratings and random-dot patterns to participants’ fingerpads with a variety of spatio-temporal properties, such as spatial period, speed, and indentation depth, then compared the psychophysical performance to known properties of mechanoreceptors and S1 neurons.

## Materials and Methods

### Participants

Ten participants (six men and four women, aged 23–32 years) were tested in all three experiments. All participants reported normal tactile sensation and had no systemic or neurological diseases. The experimental protocol was reviewed and approved by the Institutional Review Board of Chang Gung Memorial Hospital.

### Apparatus

**FIGURE 1:**
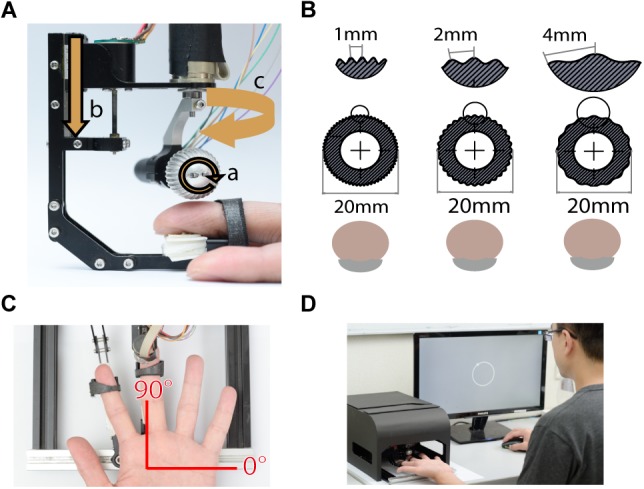
Experimental apparatus. **(A)** The miniature tactile stimulator with three motors, each of which controls one degree of freedom, including **(a)** rotation speed, **(b)** indentation depth, and **(c)** moving direction of the ball. **(B)** Three grating balls, with spatial periods of 1, 2, and 4 mm. The fingerpad was positioned palmar side up. **(C)** Motion stimuli were delivered to the fingerpad of the left middle finger via the stimulator. The red axes show the coordinates used for stimulus presentation and participant report. **(D)** For each trial, the motion stimulus was delivered for 1 s, and then the participant reported the perceived direction of motion by clicking the mouse on a circle on the computer display. During the experiment, the stimulator was housed inside a specially designed case, such that the participant could not see the tactile stimuli.

Tactile stimuli were presented using a miniature ball stimulator with different kinds of aluminum balls. The details of the stimulator and stimulus ball have been reported in our previous work (Pei et al., 2014). Briefly, the stimulator consists of individual units that deliver motion with three degrees of freedom: rotation to produce motion, vertical excursion to control the depth of indentation into the skin, and arm orientation to control the direction of motion (Figure 1A). The replaceable stimulus ball indents on the participant’s fingerpad and provides the motion stimulus. In this study, the stimuli were applied via 20 mm-diameter aluminum balls consisting of either gratings (Figure 1B) or random dots (Figure 6A). The grating ball was engraved with sinusoidal gratings with a spatial period of 1, 2, or 4 mm, a trough-to-peak ridge height of 250 or 500 μm, and a duty cycle (ridge width/spatial period) of 0.6. The spatial period, speed, and indentation depth used in each experiment are described in the section “Procedure.” The grating stimulus was designed to produce a distinctive direction and orientation sensation; its orientation was orthogonal to its motion direction. The random-dot ball was engraved with a random array of dots with a dot-to-dot distance of 3 ± 1 mm. Although different types of stimulators were used, the dot density and distance variance for random dot patterns matched those used in our previous study (Pei et al., 2010). The random-dot ball was designed to present motion that lacked periodic, oriented motion energy (Figure 6A).

The finger holder was adjustable to accommodate different finger lengths, and a U-shaped socket was attached to the tip of the finger holder to support the nail. The center of the stimulus ball was aligned with the center of the U-shaped socket so that the fingerpad could be positioned right below the center of the stimulus ball (Figure 1A). We defined the stimulus and reported motion direction using a coordinate system, in which 90° indicates the proximal-to-distal direction along the long axis of the digit, and 0° indicates the ulnar direction (Figure 1C).

### Procedure

On each trial, a moving tactile pattern was presented to the participant’s left middle fingerpad for 1 s. Specifically, the ball rotated and gradually indented upon the participant’s fingerpad with an indentation depth of 250 or 500 μm depending on the experimental condition. During the experiment, the stimulator was housed inside a specially designed case so that participants could not see the motion of the ball. The participants reported the perceived direction of motion using the right hand to perform a mouse click on a circle on the computer screen (Figure 1D). The inter-stimulus interval was 1 s between the participant’s response and the onset of the subsequent stimulus.

### Experiment 1: Spatio-Temporal Effect

In Experiment 1, we characterized the relationship between the spatio-temporal properties of the grating stimulus and the magnitude of IMR. The experiment was performed via block design using grating balls with spatial periods 1, 2, and 4 mm. In a factorial design, within each stimulus ball block, the stimulus was presented with a combination of directions ranging from 17.5° to 347.5° in steps of 30°, with a surface moving speed of 20, 40, 80, 160, or 320 mm/s, and with an indentation depth of 250 μm in pseudo-random order. We presented a total of 180 combinations (12 directions × 3 spatial periods × 5 speeds) with 10 repetitions. We tested each stimulus ball as a separate block, and the participant was allowed to rest between blocks.

### Experiment 2: Indentation Depth Effect

In Experiment 2, we characterized the relationship between the indentation depth of the grating ball and the magnitude of IMR. This experiment was identical to Experiment 1 except that the moving speed was 40, 80, or 160 mm/s, and the indentation depth was 250 or 500 μm. We presented a total of 216 combinations (12 directions × 3 spatial periods × 3 speeds × 2 indentation depths) with 10 repetitions.

### Experiment 3: Orientation Effect

In Experiment 3, we examined whether orientation information (i.e., the grating) is necessary for the induction of IMR. We presented directional stimuli using the random-dot ball that lacked periodic, oriented motion energy. This experiment was identical to Experiment 2 except that the stimulus was the random-dot ball. The average dot-to-dot distance was 3 ± 1 mm; dot diameter, 1 mm; and dot height, 500 μm). The speed was 40, 80, or 160 mm/s, and the indentation depth was 250 or 500 μm. We presented a total of 72 combinations (12 directions × 3 speeds × 2 indentation depths) with 10 repetitions.

### Data Analysis

#### Perceptual Bias Calculation and Bimodal von Mises Fitting

We defined perceptual bias as the angular difference between perceived and veridical directions:

(1) Perceptual bias=Perceived direction−Veridical direction

We estimated the distribution of perceptual bias across directions via a histogram binned by 15°, yielding *f*(θ*_i_*) for the perceptual bias direction θ*_i_*. We fitted this binned histogram using the bimodal von Mises function (Mardia, 1975), which is similar to circular Gaussian distribution. We defined the bimodal von Mises function as:

(2) $$f(\theta_{i})={\text{A}}_{1}{\text{e}}^{\beta_{1}\cos(\theta_{i}-\theta_{1})}+{\text{A}}_{2}{\text{e}}^{\beta_{2}\cos(\theta_{i}-(\theta_{1}+180{}^{\circ}))}+\gamma$$

**FIGURE 2:**
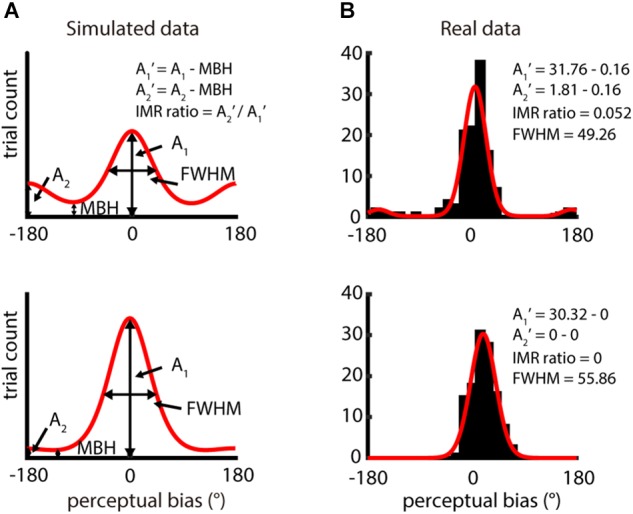
Analysis of IMR from distribution of perceptual bias – difference between perceived direction and veridical direction. **(A)** Two simulation distributions of perceptual bias generated by bimodal von Mises function. A_1_ and A_2_ reflect the amplitude of the highest peak and the opposite direction of the highest peak, respectively. A_1_′ and A_2_′ were computed by subtracting A_1_ and A_2_ with the minimal bin height (MBH) and IMR ratio = A_2_′/A_1_′. Data in the upper panel show obvious IMR, but data in the lower panel show weak IMR. **(B)** Single-participant responses from two different spatio-temporal conditions (upper panel: spatial period = 2 mm, speed = 80 mm/s; lower panel: spatial period = 4 mm, speed = 80 mm/s). The red curve is the bimodal von Mises fit for the binned perceptual bias (bin size = 15°). Results in the upper and lower panels had higher (IMR ratio = 0.052) and lower IMR (IMR ratio = 0), respectively.

For this function, the direction at the highest peak and the direction opposite to the highest peak have amplitudes A_1_ and A_2_, respectively (Figure 2A). θ*_i_* is the direction of perceptual bias, θ_1_ is the direction of A_1_, and θ_1_ + 180° is the direction of A_2_, β_1_ and β_2_ defines the width of each von Mises, and γ is the numerical minimal bin height (MBH). This function assumes that the distribution of perceptual biases peaks twice, one close to the veridical direction and the other at the opposite direction. The fitting was made using the fit function in MATLAB (MathWorks, Inc., Natick, MA, United States). The goodness-of-fit of the bimodal von Mises fitting was evaluated using *R*^2^ and root-mean-square error (RMSE) (passing criteria: *R*^2^ ≥ 0.6 or RMSE ≤ 5).

### IMR-Ratio Analysis

The magnitude of IMR was computed as the relative amplitude between A_2_′ and A_1_′. An IMR ratio of 1 indicates a condition with the highest IMR, and a ratio of 0 indicates absence of IMR:

(3) $${{\text{A}}^{\prime }}_{1}={\text{A}}_{1}-\text{MBH}$$

(4) $${{\text{A}}^{\prime }}_{2}={\text{A}}_{2}-\text{MBH}$$

(4) $$\mathrm{IMR ratio}={{\text{A}}^{\prime }}_{2}/{{\text{A}}^{\prime }}_{1}$$

where A_1_ is the amplitude of the highest peak in the fitting function and A_2_ is that of the amplitude of 180° away from A_1_. MBH is the minimum height of the distribution predicted by the bimodal von Mises fit and is computed as the height where the first derivative of the bimodal von Mises fit is 0, and the second derivative is positive. We show examples of the bimodal von Mises function fitted to both simulated data (Figure 2A) and actual data (Figure 2B). For simulated data, two peaks are 180° apart, and the base is MBH. The upper part is shown as data with higher IMR ratios, and the lower part, as data with lower IMR ratios. In the fitting function, we characterized the full width at half maximum (FWHM) of the peak of amplitude A_1_, which estimates the precision of perceived motion direction.

### Statistical Analysis

We used R (R: A language and environment for statistical computing. R Foundation for Statistical Computing, Vienna, Austria) for statistical tests. For both IMR and FWHM, we performed repeated measures ANOVA to test the effects of spatial period, speed, and spatial period × speed interaction. We corrected for multiple comparisons using the Holm–Bonferroni method (Holm, 1979). To examine whether FWHM is related to IMR, we also performed Pearson’s correlation between FWHM and IMR for each spatio-temporal condition.

To explore the effect of stimulus direction on IMR, we examined the probability to yield IMR. Specifically, we measured the proportion of trials in which the perceived direction was reversed (the range delimited by 180° perceptual bias ± 45°). In this analysis, for each spatio-temporal combination, we pooled all the probabilities of IMR across participants, and applied the Chi-square test to assess whether it was significantly different from a uniform distribution.

We also performed repeated measures ANOVA to examine whether indentation depth (250 or 500 μm) affects IMR, and whether orientation information (grating balls vs. random dot ball) affects IMR and FWHM.

## Results

### Experiment 1: Spatio-Temporal Effect

**FIGURE 3:**
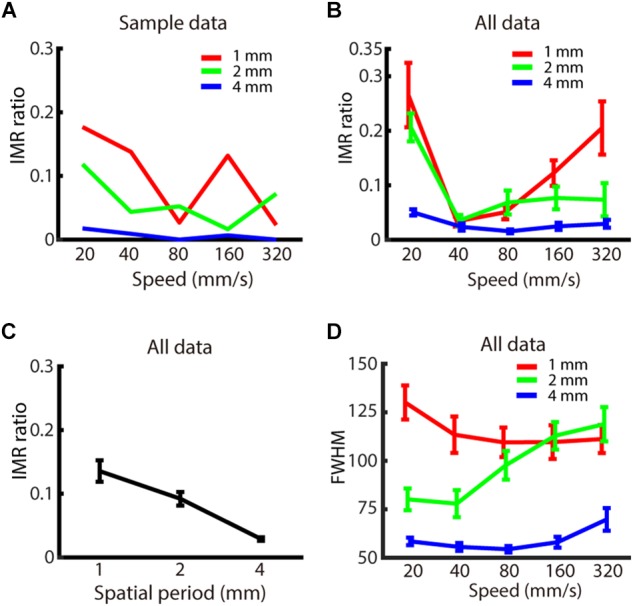
Experiment 1: Spatio-temporal dependence of IMR. **(A)** IMR ratios across spatio-temporal parameters for one participant. **(B)** IMR ratio averaged across participants as a function of spatial period and speed (red: 1 mm, green: 2 mm, blue: 4 mm). **(C)** IMR ratio as a function of grating spatial period. **(D)** FWHM of A_1_ averaged across participants as a function of spatial period and speed (red: 1 mm, green: 2 mm, blue: 4 mm). Bars indicate standard error of the mean.

We first characterized the degree to which the spatio-temporal parameters of a moving grating affect the magnitude of IMR, by measuring the probability of perceiving the opposite direction when a moving grating is presented on the participant’s left middle fingerpad. For each spatio-temporal combination, the IMR ratio was obtained by computing the relative amplitude between the transformed amplitudes of two peaks from a bimodal von Mises fit. Indeed, IMR was observed across a variety of spatio-temporal conditions (Figure 3A shows the data from a sample participant; Supplementary Figure 1 shows data in greater detail). Additionally, the goodness-of-fit for the bimodal von Mises fit passed the criteria in all participants.

We then evaluated whether the spatio-temporal parameters of spatial period and speed affected the IMR ratio (Figure 3A,B). By using two-way repeated measures ANOVA and *post hoc* analysis, we found that the IMR ratio was significantly affected by both spatial period (*F*_(2,126)_ = 6.741, *p* < 0.01) (significant pair: 1 vs. 4 mm) (Figure 3C) and speed (*F*_(4,126)_ = 4.153, *p* < 0.01) (significant pairs: 20 vs. 40 mm/s; 20 vs. 80 mm/s). We also performed linear regression for data below and above 40 mm/s, respectively. Data below 40 mm/s showing that the IMR ratio decreased as a function of speed (<40 mm/s: slope = –2.29, adjusted *R*^2^ = 0.128, *t* = −3.1, *p* < 0.01, *F*-statistics vs. constant model = 9.66, *p* < 0.01), and data above 40 mm/s showing that IMR ratio increased as a function of speed (>40 mm/s: slope = 0.123, adjusted *R*^2^ = 0.032, *t* = 2.21, *p* < 0.05, *F*-statistics vs. constant model = 4.9, *p* < 0.05). Specifically, the IMR ratio was highest in conditions with a smaller spatial period and extreme speed (such as 20 and 320 mm/s). However, the interaction between spatial period and speed was not statistically significant (*F*_(8,126)_ = 0.986, *p* = 0.450).

We also characterized the FWHM, which estimates the precision of perceived motion direction. FWHM of A_1_ (Figure 3D) was significantly affected by spatial period (*F*_(2,126)_ = 41.745, *p* < 0.001) (significant pairs: 1 vs. 2 mm; 1 vs. 4 mm; 2 vs. 4 mm), but neither by speed (*F*_(4,126)_ = 1.372, *p* = 0.247) nor interaction between spatial period and speed (*F*_(8,126)_ = 1.683, *p* = 0.109). For the comparison between IMR and FWHM of A_1_ across spatio-temporal combinations, only 1 out of 15 combinations showed a significant correlation (4 mm, 320 mm/s: *R*^2^ = 0.670, *t* = 4.02, *p* < 0.01). However, there was a similar trend for both IMR and FWHM in a spatial period, both of which showed lower values in 4 mm compared with 1 and 2 mm conditions.

**FIGURE 4:**
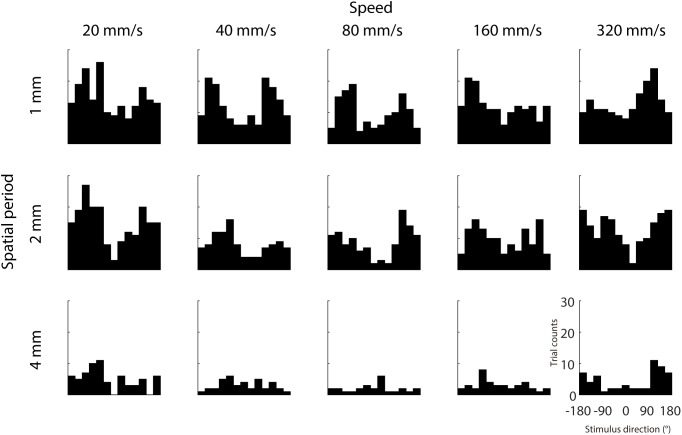
Dependence of IMR on stimulus direction. We analyzed the distribution of stimulus directions that were putatively assigned to the IMR, by counting the trials with stimulus direction within range delimited by the range delimited by 180° perceptual bias ± 45°. Trial counts of IMR as a function of stimulus direction from each spatio-temporal combination. IMR tended to occur in certain stimulus directions (around 120° and –120°) and spatio-temporal combinations.

We further examined whether IMR is more commonly observed in specific directions and less in other directions, which might be consistent with the oblique effect found in visual and somatosensory domains (Essock et al., 1992, 1997; Eves and Novak, 1998). Results across participants showed that IMR tended to occur in certain stimulus directions (around 120° and –120°) and spatio-temporal combinations (Figure 4). This IMR probability is non-uniformly distributed across stimulus directions in a majority of spatio-temporal combinations, as 8 out of 15 combinations had distributions significantly different from a uniform distribution (Chi-square test for uniformity, *p* < 0.05).

### Experiment 2: Indentation Depth Effect

**FIGURE 5:**
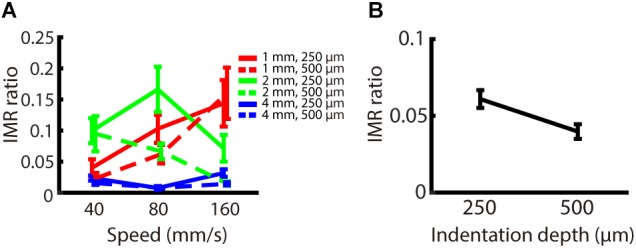
Experiment 2: Effect of indentation depth on IMR. The experimental paradigm was almost identical to Experiment 1, except that we presented speeds only in the middle-speed ranges and indentation depth as 250 or 500 μm. **(A)** IMR ratio under spatio-temporal (spatial period; red: 1 mm, green: 2 mm, blue: 4 mm) and indentation depth (solid lines 250 μm, dashed lines 500 μm) manipulations. **(B)** IMR ratio as a function of indentation depth showed that IMR ratio did not significantly depend on indentation depth (null hypothesis test *F*_(1,162)_ = 1.855, *p* = 0.175). The equivalence test also confirmed that there was no significant difference in the IMR ratio between indentation depths (Welch two sampled TOST equivalence test, boundaries = ±0.1886, *DF* = 143.14, *p* < 0.001). Bars indicate standard error of the mean.

The indentation depth of the stimulus has been shown to be correlated with the signal strength of SA1 and RA inputs (Johnson and Hsiao, 1992). We found that, across participants, the indentation depth did not alter the strength of IMR across these spatio-temporal ranges (Figure 5A), as reflected by the fact that the IMR ratio did not significantly depend on indentation depth (null hypothesis test *F*_(1,162)_ = 1.855, *p* = 0.175). The equivalence test also confirmed that there was no significant difference in the IMR ratio between indentation depths (Welch two sampled TOST equivalence test, boundaries = ±0.1886, *DF* = 143.14, *p* < 0.001) (Figure 5B).

### Experiment 3: Orientation Effect

**FIGURE 6:**
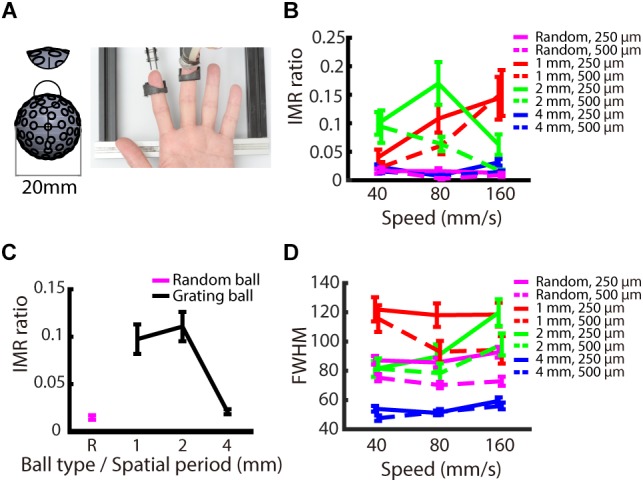
Experiment 3: Orientation dependence on IMR. To examine whether orientation information is necessary to induce IMR, we presented a random-dot pattern ball. **(A)** Experimental apparatus. The random-dot pattern ball had dots randomly arranged on the ball surface with the average dot-to-dot distance of 3 ± 1 mm. The miniature ball stimulator and hand position were the same as those in Experiments 1 and 2. **(B)** IMR ratios across different ball types, including spatial period, speed, and indentation depth combinations. **(C)** The random-dot pattern induced the lowest IMR ratio compared with the grating balls for the spatial periods of 1 and 2 mm (1 mm vs. random-dot, *F*_(1,216)_ = 15.125, *p* < 0.001; 2 mm vs. random-dot, *F*_(1,216)_ = 13.530, *p* < 0.01) but not for gratings with spatial period of 4 mm (4 mm vs. random-dot, *F*_(1,216)_ = 0.049, *p* = 1). **(D)** FWHM across different ball types, including spatial period, speed, and indentation depth combinations. Bars indicate standard error of the mean.

We examined whether the IMR occurs only for oriented stimuli, such as gratings, or if it is also observed for stimuli without orientation information. The logic behind this condition is that IMR will be orientation dependent if its mechanism is mediated by orientation-selective neurons in the somatosensory cortex. We presented stimuli with random-dot patterns to participants’ fingerpads (Figure 6A) and compared the results to those obtained using the grating stimuli. The results showed that the IMR ratio was relatively low in the random-dot ball condition (Figure 6B). Additionally, the IMR ratio of the random-dot pattern was significantly lower than those of the grating patterns with smaller spatial periods (1 and 2 mm) (*post hoc* of repeated measures ANOVA: 1 mm vs. random-dot, *F*_(1,216)_ = 15.125, *p* < 0.001; 2 mm vs. random-dot, *F*_(1,216)_ = 13.530, *p* < 0.01) but not for grating with a spatial period of 4 mm (4 mm vs. random-dot, *F*_(1,216)_ = 0.049, *p* = 1) (Figure 6C). Additional analyses showed that the FWHM of the random-dot ball was narrower than that of the 1 mm grating ball (*post hoc* of repeated measures ANOVA: 1 mm vs. random-dot, *F*_(1,216)_ = 26.803, *p* < 0.001), wider than that of the 4 mm grating ball (4 mm vs. random-dot, *F*_(1,216)_ = 22.842, *p* < 0.001), and not significantly different from that of the 2 mm grating ball (2 mm vs. random-dot, *F*_(1,216)_ = 3.552, *p* = 0.059). These findings indicate that the relatively low IMR ratio of the random-dot ball was not simply a result of better precision (Figure 6D), suggesting that edge orientation might be a major contributor to IMR.

## Discussion

We characterized tactile IMR using grating stimuli across a combination of spatio-temporal, indentation depth, and shape conditions. We found that the IMR perception was stronger for smaller spatial periods and for extreme (low and high) surface speeds, but no evidence was found for the interaction between the spatial period and speed. IMR tended to occur when a grating with motion direction of around 120° and –120° was presented, revealing a strong directional anisotropy for IMR. IMR was not altered by indentation depth, indicating its stability at this range of stimulus intensities. Finally, IMR was much weaker for random-dot stimuli, suggesting that periodic, oriented motion energy might be an important contributor to IMR. Indeed, these results are consistent with the motion processing mechanisms previously proposed by Pei et al. (2008, 2010, 2011). That is, a majority of motion-selective units also have orientation selectivity, suggesting that orientation and direction processing overlap in S1 (Pei and Bensmaia, 2014). In this study, the existence of IMR to tactile gratings, and the need for stimulus orientation to elicit stronger tactile IMR, further support the notion that motion, and spatial periodicity are both used in the processing of motion direction.

The effect of spatial period on IMR could be due, in part, to tactile acuity. A previous report using the Johnson–Van Boven–Philips (JVP) dome grating (Van Boven and Johnson, 1994), a gold standard of tactile acuity assessment for orientation discrimination, showed that the threshold for the left middle fingerpad was 1.51 mm (range, 0.8–2.5 mm) in healthy participants (Vega-Bermudez and Johnson, 2001). In our study, a higher IMR was observed when spatial periods were 1 and 2 mm, a finding that could be explained by the higher IMR when the spatial period is close to the threshold of tactile acuity.

Both visual and tactile acuity for orientation discrimination is associated with the known properties of sensory neurons (Lamme et al., 1994; Van Boven and Johnson, 1994; Craig and Kisner, 1998; Craig, 1999; Geisler et al., 2001), which shows support for these similarities between the two systems. Visual motion information is first processed in the retina as the spatio-temporal changes in luminance and contrast. This information is later linearly rectified and temporally summed in the cortex to compute motion direction (Wassle, 2004). The pathway involves the primary visual cortex (V1), middle temporal cortex (MT), the medial superior temporal cortex (MST), and other parietal lobe areas (Van Essen and Manusell, 1983). The wagon wheel illusion was first described clearly as a visual IMR by Purves et al. (1996). Levichkina et al. (2014) reported that in the visual wagon wheel illusion, spatial proximity rather than temporal frequency determines the strength of visual IMR. Holcombe and Seizova-Cajic (2008) compared IMR for visual, proprioception, and touch, and found that both proprioceptive and tactile stimuli can induce IMR. We demonstrated the existence of tactile IMR and showed that it was influenced by spatial period and speed, but not by the interaction between spatial period and speed. This shows that IMR is not strongly linked to the temporal frequency properties processed by specific mechanoreceptors, which suggests that IMR may instead be processed at the cortical level.

We found that the IMR was strongest at extreme speeds. Essick and Whitsel (1985) used brush stimuli to test human participants in discriminating cutaneous motion direction under different speed conditions, and found that discrimination is strongest when stimuli move at 74–201 mm/s, and weakest at 11 and 1484 mm/s. For human tactile speed detection, Cybulska-Klosowicz et al. (2011) tested speeds ranging from 50 to ∼1000 mm/s and found a decrease in performance at higher speeds (>200 mm/s). A majority of S1 neurons have a monotonically increasing spiking rate as moving speed increases (Tremblay et al., 1996; Depeault et al., 2013). We previously reported that, across the neuronal population, some neurons have direction selectivity peaking at a specific speed whereas other neurons have monotonically increasing or decreasing direction selectivity as the speed increases from 10 to 80 mm/s (Pei et al., 2010). These findings cannot directly explain why IMR was mostly observed at extreme speeds. We speculate that neuronal processing for motion direction is optimized in the middle ranges so that IMR, which is a reflection of ambiguity in perceiving motion direction, is more frequent when the stimulus property is outside the optimized ranges.

Previously, discrimination of perceived tactile motion direction was reported to be more precise at certain orientations, with its best performance at the proximal–distal orientation, a phenomenon called tactile anisotropy (Essock et al., 1992, 1997; Olausson and Norrsell, 1993; Keyson and Moutsma, 1995; Drewing et al., 2005). Here we found stronger tactile IMR (inability to discriminate two motion directions for certain stimulus orientation) when a stimulus motion direction of around 120° and –120° was presented, a directional property (IMR anisotropy) that has not been previously reported in touch.

The miniature ball stimulator used in this study elicits both spatio-temporal patterns and shear forces (Olausson and Norrsell, 1993; Essick, 1998; Nakazawa et al., 2000; Pei and Bensmaia, 2014). Seizova-Cajic et al. (2014) found that opposing lateral shear information processed by SA2 influences directional discrimination. However, in our experimental design, the lateral shear force was congruent with motion direction, suggesting that it was not the main contributor to IMR anisotropy. IMR anisotropy might be related to inhomogeneous spatio-temporal information processing at the cortical level, such as the elongated cortical representation for fingers in primate S1 (Kaas, 1991).

Another hypothesis for IMR is motion adaption. Barlow and Hill (1963) found a gradual decrease in neuronal activity when rabbit retinal ganglion cells were stimulated by a rotating random pattern for 15–20 s. Furthermore, the neuronal responses recovered fully 30 s after stopping the stimulus motion. For the middle temporal cortex, a higher visual cortical area, induction time for adaptation to visual motion has been reported at a much longer timescale of 28 s (Tolias et al., 2001). This time course was analogous to that of visual IMR in human psychophysical experiments (Kline et al., 2004; Sokoliuk and VanRullen, 2013). Motion adaption might suppress the responses of early-stage direction-selective motion detectors, so that other detectors tuned to the opposite direction become more sensitive to variations in the input signals. As proposed by Kline et al. (2004) and other reports (Kline et al., 2004; Holcombe and Seizova-Cajic, 2008; Kline and Eagleman, 2008), it is speculated that IMR may be manifested by such adaptive “uncovering” of neural activity encoding the opposite direction.

For tactile motion aftereffect or adaptation experiments, researchers have tested long stimulus durations, but the exact minimum required time has not been examined. Watanabe et al. (2007) adopted a 10 s adaptation time using vibrotactile stimulation to induce a motion aftereffect, while McIntyre et al. (2012, 2016b) tested a 30-s adaptation time using a cylinder drum with a variety of ridged rubber surface stimulations to induce speed adaptation, and used a 3 or 10 s adaptation time using tactile pin array to induce the motion-direction aftereffect (McIntyre et al., 2016a). For tactile–visual cross-modal interaction, Konkle et al. (2009) used a 10 s adaption time to induce a motion aftereffect for the perceived direction. We found that tactile IMR occurred immediately after stimulus onset and that there was no cumulative effect over trials (Supplementary Figure 2). If adaption mechanisms underlie tactile IMR in our experiments, they must happen much more quickly, within 2 s.

The next question is which neural substrates in the processing pathway should underlie tactile IMR. Some have proposed that visual IMR can only be induced to periodic stimulus patterns (Purves et al., 1996; Simpson et al., 2005; VanRullen et al., 2005), but Kline and Eagleman (2008) demonstrated that a non-periodic random-dot pattern could also induce IMR. We found that a smaller spatial period grating in some speed ranges elicited a higher tactile IMR, whereas the random-dot pattern elicited a much weaker tactile IMR, implying that orientation information might contribute more to tactile IMR than the random-dot pattern used here, which lacked strong periodic, oriented motion energy. We applied gratings such that perceived directions of both motion and IMR were orthogonal to the moving edge (Pei et al., 2008). Future studies might apply barber-poles, whose moving edges have a variety of relative orientation angles with respect to motion direction (Bicchi et al., 2003), to examine the effect of edge orientation on IMR.

## Conclusion

In summary, we demonstrated robust tactile IMR among the participants. Our results shed light on the possible neural substrates underlying tactile IMR, and revealed that tactile IMR has a directional preference, an IMR anisotropy that may be related to previously reported tactile anisotropy in directional discrimination. Finally, we found that gratings elicited a higher tactile IMR, whereas the random-dot pattern we used elicited a much weaker tactile IMR, indicating that orientation information might contribute more to tactile IMR. Considering a previous report that visual IMR could also be elicited by non-periodic stimuli (Kline and Eagleman, 2008), it is possible that the lack of IMR to our random-dot pattern could be due to the weakness of the periodic, oriented motion energy in the specific pattern we tested. To resolve this issue, we may try other random dot patterns in the future. The requirement for oriented stimuli for tactile IMR suggests that the underlying neural mechanisms are mediated by orientation-selective neurons in the somatosensory pathway.

## Ethics Statement

All testing procedures were approved by the Institutional Review Board for Human Research of the Chang Gung Memorial Foundation. The participants provided written informed consent and were paid for their participation.

## Author Contributions

Y-CH, C-IY, J-JH, C-HH, CH, and Y-CP designed the project, organized the research, and wrote the manuscript. Y-CH and C-HH performed the experiments. Y-CH analyzed the data.

## Conflict of Interest Statement

The authors declare that the research was conducted in the absence of any commercial or financial relationships that could be construed as a potential conflict of interest.
