# Supplementary figures and images for: Illusory Motion Reversal in Touch
Source: Front Neurosci. 2019 Jun 14;13:605. doi: 10.3389/fnins.2019.00605 (PMC6587367; doi:10.3389/fnins.2019.00605)

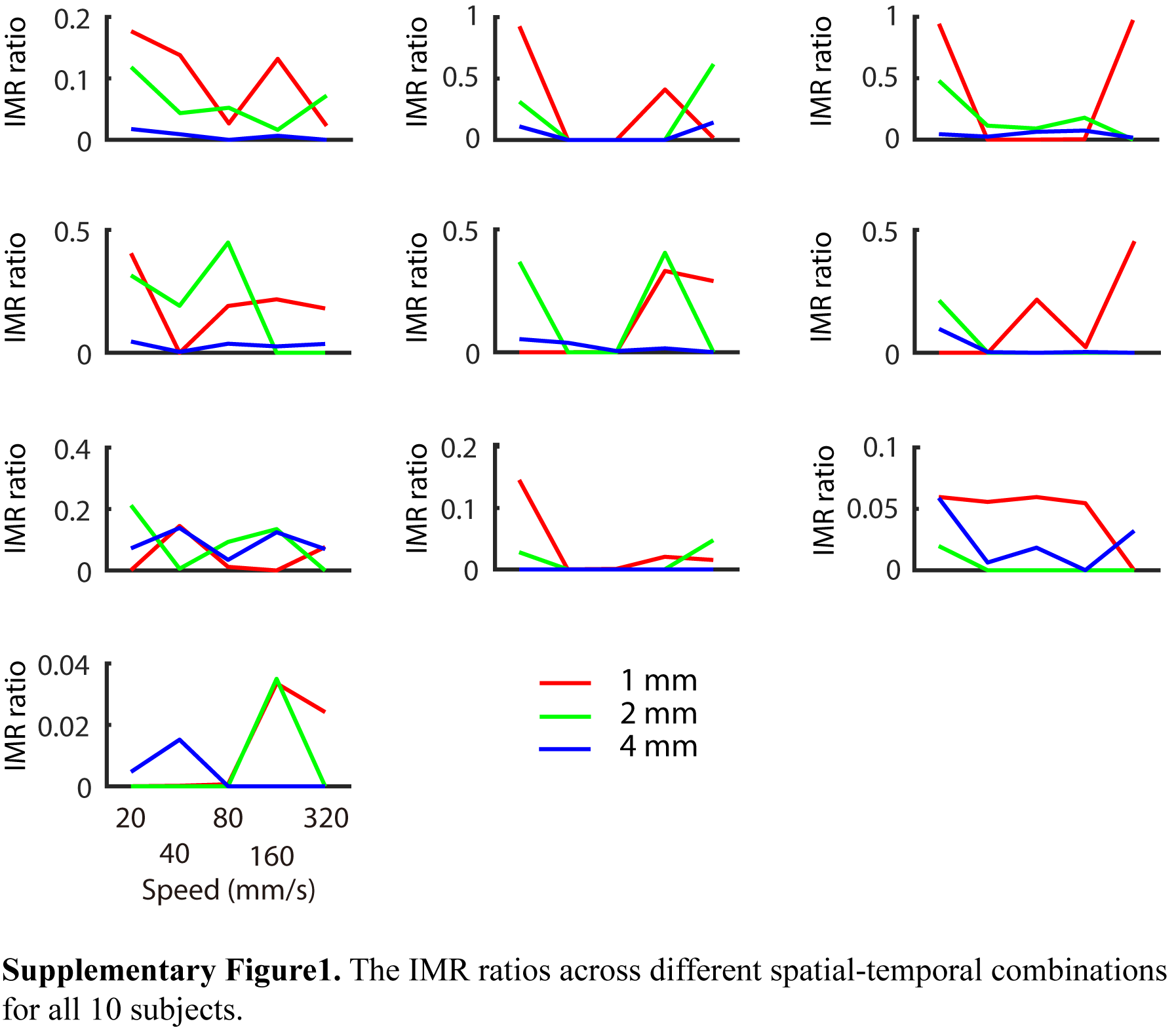

Supplement: Supplementary file 1 [file Image_1.TIF]

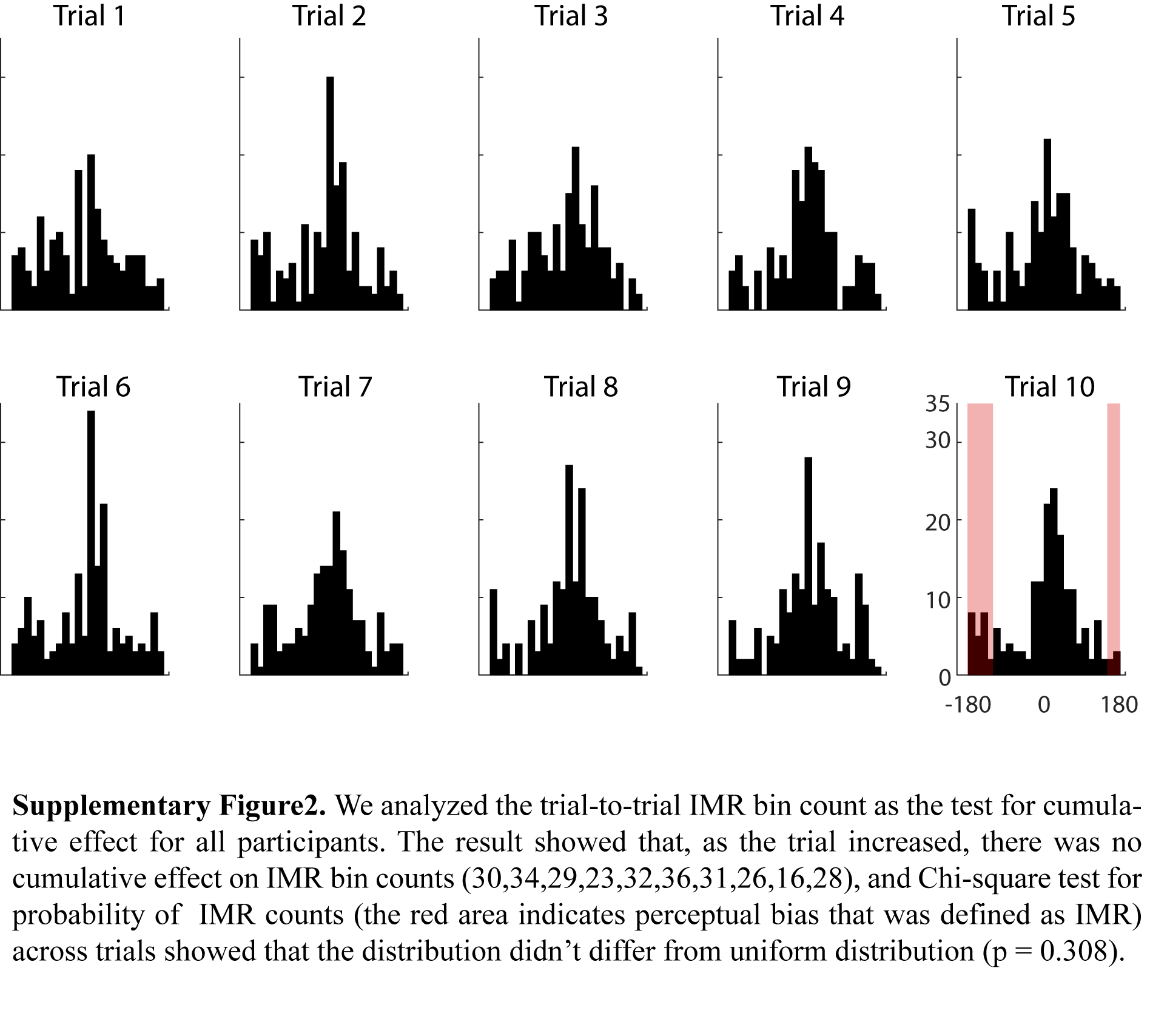

Supplement: Supplementary file 2 [file Image_2.TIF]
